# Immune Negative Regulator TIPE2 Inhibits Cervical Squamous Cancer Progression Through Erk1/2 Signaling

**DOI:** 10.1515/biol-2019-0059

**Published:** 2019-12-31

**Authors:** Li-Qiong Huang, Bo Zheng, Yi He

**Affiliations:** 1 Department of Obstetrics and Gynecology, Xianning Central Hospital, The First Affiliated Hospital of Hubei University of Science and Technology, 228 Jingui Road, Xianning 437100, China

**Keywords:** cervical squamous cancer, tumor necrosis factor-α-induced protein-8-like 2, proliferation, invasion, extracellular signal-regulated kinase

## Abstract

Tumor necrosis factor (TNF)-α-induced protein-8-like 2, or TIPE2, is a newly found immune negative regulatory molecule. This study further investigated the role of TIPE2 on proliferation and invasion of cervical squamous cancer cells. Expression of TIPE2 was compared in cervical squamous cancer tissues and adjacent normal tissues by Western blot and immunohistochemistry (IHC). Cervical squamous cancer cell lines, SiHa and C33A, were transfected with recombinant plasmid encoding TIPE2 and tested for cytologic characteristics. The impact of TIPE2 on phosphorylation of extracellular signal-regulated kinase (Erk) signaling pathway was also tested by Western blot analysis of key factors. TIPE2 expression was higher in cervical cancer tissues than that in normal tissue. IHC score of tumor tissue was negatively associated with lymphatic metastasis. Over expression of TIPE2 effectively inhibited the proliferation of cervical cancer cells. Wound healing and transwell assay showed that over expression of TIPE2 suppressed cell migration and invasion in vitro. Meanwhile, phosphorylation of Erk1/2 and upstream mitogen-activated protein kinase kinase (MEK) 1/2 was reduced by TIPE2. TIPE2 is negatively related with development of cervical squamous cancer. TIPE2 is an inhibitory factor of proliferation and invasion of cervical squamous cancer cells, probably through inhibiting Erk signaling pathway.

## 1 Introduction

Cervical cancer is one of the most common malignant tumors of female genital tract, and the mortality rate is higher than 1/10^4^ in underdeveloped areas such as Africa and South-Eastern Asia [1]. The incidence and mortality of cervical in China showed a significant increasing trend in recent years [2]. Understanding the pathogenesis of cervical cancer and exploring new treatment methods are of great significance to reduce the burden of cervical cancer. The pathogenesis of cervical cancer is complex, involving abnormal expression of multiple oncogenes or tumor suppressor genes. The destruction of immune dynamic balance is an important mechanism of tumorigenesis and immune-based cancer therapies have gained more and more attentions [3].

Tumor necrosis factor (TNF)-α-induced protein-8-like 2, or TIPE2, was firstly found as an immune negative regulatory molecule in mouse model of experimental autoimmune encephalomyelitis [4, 5]. TIPE2 belongs to the TIPE family, which is a death effect domain (DED)-containing protein family [6]. TIPE2 plays a vital role in maintaining immune homeostasis and its expression is usually down-regulated in patients with infections or immune disorders [7, 8]. Murine TIPE2 is preferentially expressed in immune cells such as lymphoid or myeloid cells; while human TIPE2 can be expressed in a variety of cells, including hepatocytes, neurons, squamous epithelial cells of the cervix, and glandular epithelial cells in stomach [9].

Recently TIPE2 is gaining more attention as a novel inhibitor of tumors. The discovery of abnormal expression of TIPE2 in tumors suggests that TIPE2 may have the potential of tumor markers and therapeutic targets. TIPE2 can inhibit activity of oncogenic Ras and therefore suppress cell survival and motility of hepatocellular carcinoma [10, 11]. TIPE2 inhibits proliferation of gastric cancer cells by up-regulating p27 [12]. In renal cell carcinoma, over expression of TIPE2 is correlated with TNM stages [13]. In a recent study about intergrinαVβ6, TIPE2 expression was found to be lower in cervical cancer tissues and cervical benign lesions than in healthy cervical tissues [14]. The effect of TIPE2 on development of cervical cancer is largely unclear. Therefore, we investigated the role and possible molecular mechanism of TIPE2 on proliferation and invasion of cervical squamous cancer cells.

## 2 Materials and methods

### 2.1 Tissue samples

Cervical squamous cancer tissues and adjacent normal tissues from 40 patients were collected from the Department of Obstetrics and Gynecology, Xianning Central Hospital from January 2012 to December 2015. All the specimens were confirmed by pathological analysis. All the patients underwent operation at our hospital and none received chemotherapy or radiotherapy before sample collection. Tissue samples were surgically removed, immediately frozen in liquid nitrogen and then stored at -80°C for subsequent molecular assays.

**Informed consent**: Informed consent has been obtained from all individuals included in this study.

**Ethical approval**: The research related to human use has been complied with all the relevant national regulations, institutional policies and in accordance the tenets of the Helsinki Declaration, and has been approved by the institutional ethics committee of Xianning Central Hospital.

### 2.2 Immunohistochemistry (IHC)

A small piece of each tissue sample was fixed in 10% formaldehyde, embedded in paraffin, and cut into thin sections (5 μm thick). Standard IHC procedure was performed to detect TIPE2 expression using SABC-AP (human IgG) kit (Boster Biological Technology, Wuhan, China). The primary antibody used was synthesized anti-TNFAIP8L2 antibody (Boster, catalog #BA3300) at 1:100 dilution. The secondary antibody used was rabbit anti-human IgG-biotin (1:1000) provided by the IHC kit. Hybridization signals were colored by BCIP/NBT solution provided by the IHC kit and examined under light microscope (Olympus, Tokyo, Japan). The stained IHC slides were independently evaluated by two pathologists based on staining intensity and percentage area of positive stain as described [8]. For each slide, 5 randomly chosen fields in ×200 magnification were analyzed.

### 2.3 Cell culture

Cervical squamous cancer cell lines SiHa and C33A (American Type Culture Collection, Manassas, VA, USA) were used for *in vitro* assays. Cells were maintained in DMEM supplemented with 10% fetal bovine serum and 1% penicillin/streptomycin at 37°C in 5% CO_2_ atmosphere. Culture medium was purchased from Gibco (Thermo Fisher Scientific Inc., Carlsbad, CA, USA).

### 2.4 Plasmid construction and transfection

Full-length human TIPE2 was cloned from cDNA template, sequenced, and cloned into pRK5 vector to form pRK5-TIPE2 as described [15]. The plasmid construction was finished by Sangon Biotechnology Inc. (Shanghai, China). To endogenously over-express TIPE2, cervical cancer cells (1×106) were cultured to 80% confluency and transfected with 100 nM of pRK5-TIPE2 using Lipofectamine 2000 reagent (Invitrogen by Thermo Fisher).

### 2.5 RNA extraction and real-time quantitative PCR

Total RNA was extracted from cells using TRIzol reagent (Invitrogen by Thermo Fisher Scientific, Carlsbad, CA, USA) and was reverse transcribed into cDNA using PrimeScript RT kit (Takara Biomedical Technology, Beijing, China). Real-time quantitative PCR was performed using SYBR Green Premix Ex Taq (Takara) on Applied Biosystem 7900 PCR systems (Applied Biosystem by Thermo Fisher Scientific, Bedford, MA, USA). The thermocycling conditions were set as: 95°C for 30 sec; 40 cycles of 95°C for 5 sec and 60°C for 30 sec. The primer sequences used were as follows: sense 5’-ACTGAGTAAGATGGCGGGTCG-3’ and anti-sense 5’-TTCTGGCGAAAGCGGGTAG-3’ for TIPE2; sense 5’-GAAGGTCGGAGTCAACGGATTT-3’ and anti-sense 5’-CCTGGAAGATGGTGATGGGATT-3’ for GAPDH. Relative TIPE2 gene level was normalized to GAPDH by the 2^-ΔΔCt^ method [16].

### 2.6 Cell proliferation

Cells were harvested 48 hours after transfection, made into cell suspension (1×104/mL), and inoculated in 96-well plates (100 μL/well). During the next 5 days, the daily growth of cells was detected by CCK-8 method (Enhanced Cell Counting Kit-8, Beyotime Biotechnology, Shanghai, China). Each well was added 10 μL of CCK-8 reagent and incubated at 37°C for 1 hour. The absorbance at 450 nm was measured by microplate reader (Bio-Rad, Hercules, CA, USA).

### 2.7 Wound healing for cell migration

Cells were harvested 48 hours after transfection, made into cell suspension (2×105/mL), and inoculated in 6-well plates (2 mL/well) to form monolayer. The cell layer was gently scratched with a pipette tip and cultured with DMEM+FBS for 24 hours. The cells were observed under a light microscope at 0 hour and 24 hour to compare the injury width.

### 2.8 Transwell assay for cell invasion

Cell invasion ability was analyzed in transwell chamber (Corning by Merck, Corning, NY, USA) with 8-μm pore size polycarbonate membrane. The upper chamber was precoated with 100 μL Matrigel (1:3 dilution with serum-free DMEM). After 48 hours of transfection, cells were cultured with serum-free basic medium for 1 day before the experiment. On the day of the experiment, cells were prepared into suspension (5×105/mL) with serum-free medium, inoculated into upper chamber (200 μL), and put in 24-well plate filled with 600 μL of DMEM+FBS. After 24 hours of incubation at 37°C, cells remained in the upper chamber were gently interrupted with a wet cotton swab. The upper chamber was then fixed in methanol for 15 min, dried at room temperature, and stained with 0.1% crystal violet for 20 min. The migrating cells were counted under a light microscope at 200×magniciation. For each well, five random fields were counted.

### 2.9 Western blot

After 48 hours of transfection, cells were collected and suspended in 100 μL of RIPA lysis buffer with 1 mM PMSF (Beyotime). After incubation on ice for 10min, cell suspension was centrifuged (12000 rpm, 4°C) for 30 min to collect supernatant. The protein concentration of supernatant was determined by BCA reagent (Beyotime). Approximately 50 μg of proteins were mixed with SDS-PAGE loading buffer (Beyotime), boiled for 5 min, and separated by 10% SDS-polyacrylamide gel electrophoresis. Protein bands were then transferred onto PVDF membranes. After blocking with 5% non-fat milk for 2 hours, membranes were probed with appropriate primary antibodies overnight at 4°C. The following antibodies were used: rabbit polyclonal antibody to mitogen-activated protein kinase kinase (MEK) 1/2 (ab178876, 1:10000), rabbit polyclonal antibody to MEK1 (phospho S298) (ab96379, 1:2000), rabbit polyclonal antibody to MEK2 (phospho T394) (ab30622, 1:1000), rabbit polyclonal antibody to extracellular signal-regulated kinase (Erk) 1/2 (ab17942,1:1000), rabbit polyclonal antibody to Erk1 (phospho Y204)/Erk2 (phospho Y187) (ab47339, 1:1000), rabbit polyclonal antibody to TIPE2 (ab110389, 1:1000), rabbit monoclonal antibody to GAPDH (ab181602, 1:5000). The next, membranes were probed with goat anti-rabbit IgG H&L (horseradish peroxidase-conjugated) (ab6721, 1:5000) at room temperature for 1 hour. All the antibodies were purchased from Abcam (Cambridge, UK). Protein signals were visualized by enhanced chemiluminescence kit (Beyotime) and analyzed by Image J software.

### 2.10 Statistical analysis

Data were analyzed with SPSS software version 19.0 (SPSS Inc., Chicago, IL, USA). All cellular experiments and Western blot of tissues samples were repeated three times. Continuous variables are presented as mean ± standard deviation (SD). Classified variables between the two groups were compared by Chi-square test. Continuous variables of two groups were compared by Student’s *t* test; continuous variables among multiple groups were compared by single factor ANOVA followed by Tukey’s test. All statistical analyses were two-sided and *P* value <0.05 was considered statistically significant.

## 3 Results

### 3.1 TIPE2 is down-regulated in cervical squamous cancer tissues

**Figure 1:**
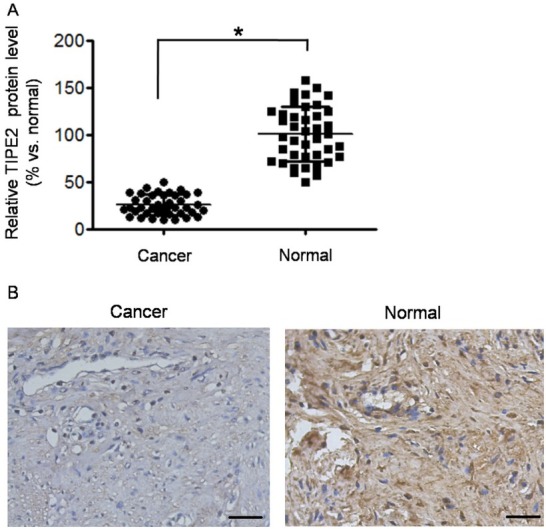
TIPE2 was down-regulated in cervical squamous cancer tissues. (A) Relative TIPE2 protein level (represented as % vs. normal) was detected by Western blot. The Western blot was repeated three times. *P<0.05 vs. normal. (B) Typical images of TIPE2 detection by immunohistochemistry (×200 magnification). The images showed medium differentiated squamous carcinoma and normal cervical tissue. Scale bar = 50 μm.

As shown in Figure 1A, the protein level of TIPE2 was significantly lower in cancer tissues than in adjacent normal tissue (*P*<0.01). IHC analysis (typical IHC images in Figure 1B) also showed that the positive expression rate of

TIPE2 in cancer tissues was significantly lower than that in normal tissues (57.5% vs. 87.5%, χ*2*=9.028, *P*=0.003). Then we analyzed the relationship between IHC scores of TIPE2 and clinical characteristics in cervical cancer tissues. As shown in Table 1, there was no significant correlation of TIPE2 score to age, differentiation grade, and FIGO stage (all *P*>0.05). However, the TIPE2 score was associated with lymphatic metastasis (*P*=0.014).

**Table 1:** Relationship between TIPE2 protein expression and clinical pathological characteristics in cervical cancer tissues

Parameters	Pathological score of TIPE2		χ^2^	P value
	0, +1	+2, +3		
**Age**				
<50	8	5	0.333	0.564
≥50	14	13		
**Differentiation grade**				
GI+GII	7	11	3.432	0.064
GIII	15	7		
**Lymphatic metastasis**				
No	5	11	6.077	0.014
Yes	17	7		
**FIGO stage**				
I-II	9	10	0.852	0.356
III-IV	13	8		

### 3.2 TIPE2 inhibits proliferation, migration, and invasion of cervical cancer cells

**Figure 2:**
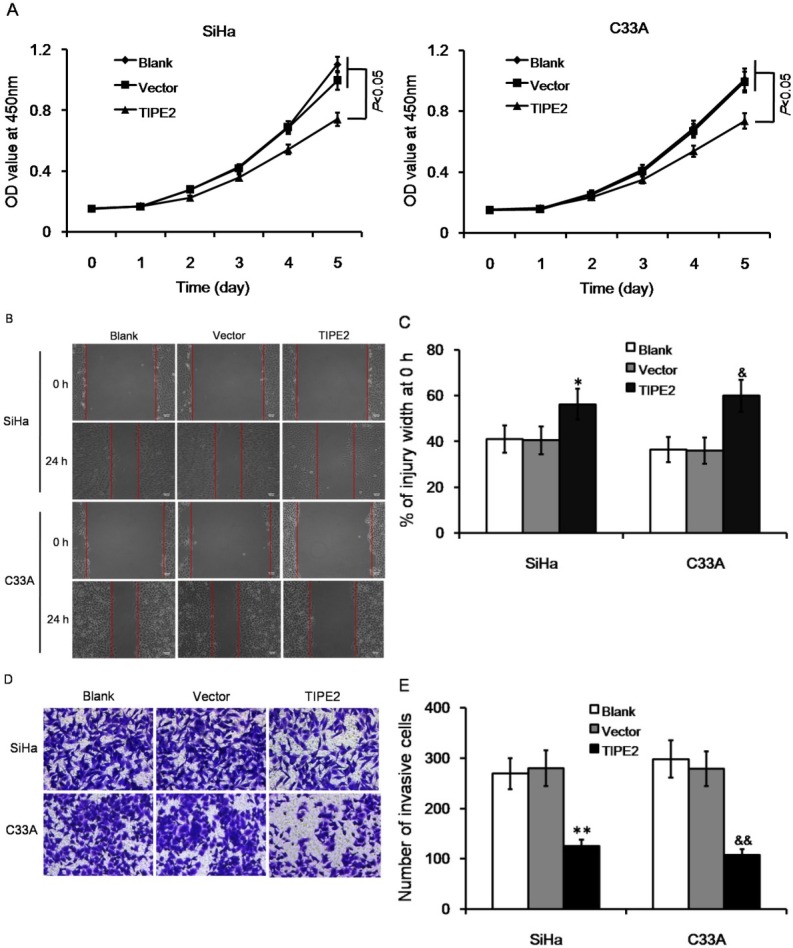
Effect of TIPE2 level on biological characteristics of cervical cancer cells without transfection (blank), transfected with empty vector (vector), or transfected with pPK5-TIPE2 (TIPE2). (A) Over expression of TIPE2 inhibited cell viability of SiHa and C33A cells. (B-C) Over expression of TIPE2 suppressed cell migration in wound healing assay. (D-E) Over expression of TIPE2 reduced number of invasive cells in transwell assay. *P<0.05 vs. blank of SiHa; **P<0.01 vs. blank of SiHa; ^&^*P*<0.05 vs. blank of C33A; ^&&^*P*<0.01 vs. blank of C33A.

**Figure S1:**
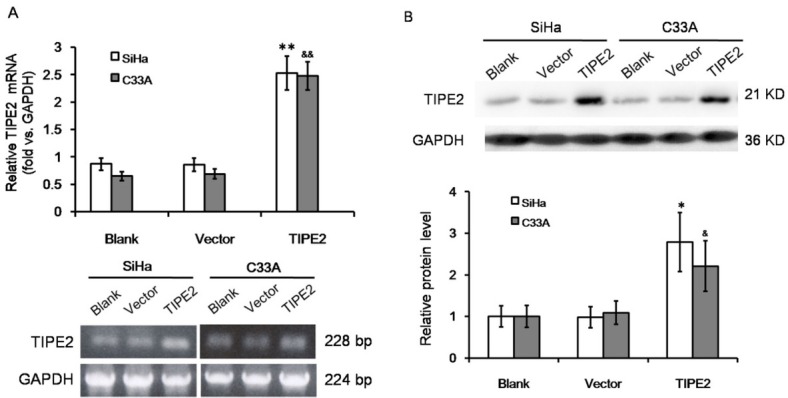
Over expression of TIPE2 in cervical squamous cancer cells by transfection. (A) RT-PCR amplification and agarose gel electrophoresis of TIPE2 mRNA level in SiHa and C33A cells without transfection (blank), transfected with empty vector (vector), or transfected with pPK5-TIPE2 (TIPE2). (B) Western blot analysis of TIPE2 protein level in SiHa and C33A cells. *P<0.05 vs. blank of SiHa; **P<0.01 vs. blank of SiHa; ^&^*P*<0.05 vs. blank of C33A; ^&&^*P*<0.01 vs. blank of C33A.

The effect of TIPE2 on cell metastasis of cervical cancer was further investigated *in vitro*. TIPE2 expression was detected in both SiHa and C33A cells; these cell lines were then transfected with pRK5-TIPE2 to over express TIPE2. The transfection efficiently increased TIPE2 expression both on mRNA level and on protein level (Figure S1). Over expression of TIPE2 inhibited cellular viability of both SiHa and C33A cells (Figure 2A, both *P*<0.05). For each time point, SiHa cells over-expressing TIPE2 showed delayed growth from day 2, and C33A cells over-expressing TIPE2 showed delayed growth from day 3. Wound healing assay showed that over expressing TIPE inhibited cell migration ability (Figure 2B-2C). After 24 hours of scratch, the wound width between cells in TIPE2 transfection group was 15%-25% wider than that in control group or empty vector group. High level of TIPE2 also reduced cell invasion ability (Figure 2D-2E). The number of invasive cells was significantly reduced by about 50%-60% when over expressing TIPE2. The results suggested that TIPE2 can suppress malignant phenotypes of cervical cancer cells *in vitro*.

### 3.3 TIPE2 inhibits phosphorylation of Erk1/2 pathway

**Figure 3:**
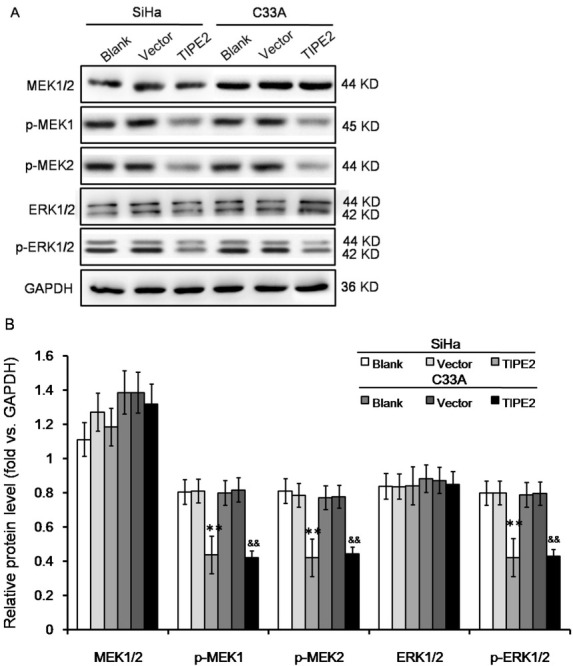
Effect of TIPE2 level on ERK signaling pathway in cervical cancer cells without transfection (blank), transfected with empty vector (vector), or transfected with pPK5-TIPE2 (TIPE2). (A-B) Western blot showed that over expression of TIPE2 inhibited phosphorylation of Erk1/2. **P<0.01 vs. blank of SiHa; ^&&^*P*<0.01 vs. blank of C33A.

Previous studies reported that TIPE2 could regulate immune responses through negative regulation of T cell receptor (TCR) pathway [5]. Classical Erk1/2 pathway is one of the main downstream pathways of TCR. Therefore, we tested the effect of TIPE2 on Erk1/2 expression. As shown in Figure 3, expression of total Erk1/2 was not changed in both SiHa and C33A cells. Meanwhile, Erk1/2 phosphorylation was reduced by TIPE2 at both the Erk1 Y204 and the Erk2 Y187. Erk1/2 is typically activated by MEK1/2. Western blot also showed that total expression of MEK1/2 was not changed, but phosphorylation was reduced at MEK1 S298 and MEK2 T394. The results suggested that TIPE2 affected phosphrylation of those factors rather than changing expression level.

## 4 Discussion

In our study, TIPE2 expression in cervical squamous cancer tissues was down-regulated compared with that in normal adjacent tissues, and was associated with lymphatic metastasis. So far only one previous paper reported that TIPE2 was down-regulated in cervical adenocarcinoma HeLa cells [17]. Another research reported that TIPE2 expression was reduced in cervical cancer tissues and cervical benign lesions [14]. Our study also explored the molecular mechanism of TIPE2 in cervical cancer. We found that TIPE2 inhibited growth, migration, and metastasis of cervical squamous cancer cells, SiHa and C33A, via suppressing phosphorylation of Erk1/2. These findings together with our findings suggested that TIPE2 should be a tumor suppressor in cervical cancer. However, the clinical diagnostic and prognostic value of TIPE2 for cervical cancer is still unclear. In our study, only 40 tumor samples were analyzed, which is not enough for other detailed studies such as stratified analysis and survival analysis.

Existing studies have shown that the expression patterns of TIPE2 in different cancers are not consistent. In addition to cervical cancer, TIPE2 also plays a tumor suppressor role in most current studies on cancers. TIPE2 up-regulated production of CD8^+^ T and natural killer (NK) cells, and inhibited breast cancer development and metastasis [18]. TIPE2 expression was lost in AGS, HGC-27, and SGC-7901 gastric cancer cells; gained-expression of TIPE2 suppresses cell migration and invasion *in vitro* via inhibiting protein kinase B (Akt)/GSK3β/β-catenin signaling [15]. TIPE2 expression was lost or reduced in primary hepatocellular carcinoma (HCC) tissues and was significantly associated with tumor metastasis, cell growth, cell migration, and cell invasion [11]. Mechanically, TIPE2 directly inhibited endogenous Rac1 and further affected extracellular matrix synthesis and angiogenesis [11]; TIPE2 could also suppress cell migration through tumor necrosis factor-α (TNF-α)-related nuclear factor-κB (NF-κB) signaling [19]; TIPE2 suppressed cell proliferation and increased the number of cells in S phase via inhibiting phosphoinositide 3-kinase (PI3K)/Akt signaling [20]. However, TIPE2 was over expressed in kidney renal cancer tissues compared with normal kidney tissues and its expression level was positively correlated with TNM staging [13]. TIPE2 was up-regulated in the cytoplasm of colon cancer tissues and HT-20 colon cancer cells by inhibiting caspase-8 activity [21]. Therefore, if TIPE2 is used as a target for development of new drugs, it may also need to consider whether specific types of cancers can benefit from treatment.

TIPE2 regulates tumorigenesis through multiple signaling pathways. In addition to the PI3K/Akt and TNF-α/NK-κB mentioned above, TIPE2 also regulates Wnt/β-catenin cascade, signal transducer and activator of transcription 3 (STAT3), and to influence proliferation, angiogenesis, apoptosis, and metastasis (for comprehensive overview see review [22]. In this study, we found that up-regulation of TIPE2 inhibited phosphorylation of Erk1/2. Similar regulational relationship was reported in hepatocellular carcinoma and gastric cancer [19, 23]. Classical mitogen-activated protein kinase (MAPK) pathway includes three branches: p38 MAPK, c-Jun N-terminal kinase (JNK), and Erk. In TIPE2 knockout murine T cells, TIPE2 is a critical regulator of JNK and p38 pathways, but ERK is not affected [5]. In other words, the regulation mechanism of TIPE2 is different in different disease models.

In conclusion, our study further showed that down-regulation of TIPE2 was associated with the development of cervical squamous cancer. TIPE2 could suppress the proliferation and migration of cancer cells by inhibiting phosphorylation of Erk1/2. Research on the molecular mechanism of TIPE2 in cervical cancer is just beginning. More extensive studies are required to elucidate the diagnostic and therapeutic values of TIPE2.
